# Supplementary material for: An empirically derived recommendation for the classification of body dysmorphic disorder: Findings from structural equation modeling
Source: PLoS One. 2020 Jun 3;15(6):e0233153. doi: 10.1371/journal.pone.0233153 (PMC7269265; doi:10.1371/journal.pone.0233153)
Supplement: S1 File — (PDF) [file pone.0233153.s001.pdf]

An empirically derived recommendation for the classification of body dysmorphic disorder: Findings from structural equation modeling

SPSS Variable	Content
gender	Gender: 1=male, 2=female
age	Age in years
sexori	Sexual orientation: 1= gay, 2 = lesbian, 3 = heterosexual, 4 = bisexual, 5 = pansexual, 6 = polysexual, 7 = asexual, 8 = other
educ	Educational attainment, 1 = without formal educational degree, 2 = vocational education, 3 = high school degree, 4 = university of applied science entrance diploma, 5 = university-entrance diploma, 6 = university of applied science diploma, 7 = university diploma
mentdis	Diagnosis of a mental disorder: 1 = yes, 0 = no
fks1, fks16 .. fks30	16 Items „Fragebogen Körperdysmorpher Symptome“ (Body Dysmorphic Symptoms Inventory); range: 0 .. 4
fks	Mean(fks1, fks16 .. fks30)
fkscut	Cutoff: fkscut = 1 if (fks15 < 3), fkscut = 0 else
edeq1 .. edeq12, edeq19 .. edeq28	22 Items “Eating Disorder Examination-Questionnaire”; range 0 .. 6
edeq	Mean(edeq1 .. edeq12, edeq19 .. edeq28)
phq1 .. phq9	9 Items „Patient Health Questionnaire”; range: 0 .. 3
phq	Mean(phq1 .. phq9)
oci1 .. oci18	18 Items „Obsessive-Compulsive Inventory-Revised“; range: 0 .. 4
oci	Mean(oci1 .. oci18)
hai1 .. hai14	14 Items “Short Health Anxiety Inventory“; range: 1 .. 5
hai	Mean(hai1 .. hai14)
h491 .. h497	7 Items “Hamburg Modules for the Assessment of Psychosocial Health”; range: 0 .. 4
h49	Mean(h491 .. h497)
lsas11 .. lsas242	48 Items „Liebowitz Social Anxiety Scale“; range: 0 .. 3
lsas	Mean(lsas11 .. lsas242)
mddi1 .. mddi13	13 Items „Muscle Dysmorphia Disorder Inventory“; range: 1 .. 5
mddi	Mean(mddi1 .. mddi13)
sps1 .. sps8	8 Items „Skin Picking Scale-Revised”; range: 0 .. 4
sps	Mean(sps1 .. sps8)
hps1 .. hps7	7 Items “Massachusetts General Hospital Hairpulling Scale”; range: 0 .. 4
hps	Mean(hps1 .. hps7)
acq1 .. acq14	14 Items “Questionnaire on Body-Related Fears, Cognitions and Avoidance: Agoraphobic Cognitions Questionnaire”; range: 1 .. 5
acq	Mean(acq1 .. acq14)

Sample size n=736; whenever required items were already inverted.
